# Differential diagnoses of fibrosing lung diseases

**DOI:** 10.1259/bjro.20190009

**Published:** 2019-06-13

**Authors:** Carolyn Horst, Bahareh Gholipour, Arjun Nair, Joseph Jacob

**Affiliations:** 1 Department of Respiratory Medicine, University College London, UK; 2 Department of Radiology, University College London, UK; 3 Centre for Medical Image Computing, University College London, UK

## Abstract

**Objectives:**

To describe the challenges inherent in diagnosing fibrosing lung diseases (FLD) on CT imaging and methodologies by which the diagnostic process may be simplified.

**Methods:**

Extensive searches in online scientific databases were performed to provide relevant and contemporary evidence that describe the current state of knowledge related to FLD diagnosis. This includes descriptions of the utility of a working diagnosis for an individual case discussed in a multidisciplinary team (MDT) setting and challenges associated with the lack of consensus guidelines for diagnosing chronic hypersensitivity pneumonitis.

**Results:**

As well as describing imaging features that indicate the presence of a fibrosing lung disease, those CT characteristics that nuance a diagnosis of the various FLDs are considered. The review also explains the essential information that a radiologist needs to convey to an MDT when reading a CT scan. Lastly, we provide some insights as to the future directions the field make take in the upcoming years.

**Conclusions:**

This review outlines the current state of FLD diagnosis and emphasizes areas where knowledge is limited, and more evidence is required. Fundamentally, however, it provides a guide for radiologists when tackling CT imaging in a patient with FLD.

**Advances in knowledge:**

This review encompasses advice from recent guideline statements and evidence from the latest studies in FLD to provide an up-to-date manual for radiologists to aid the diagnosis of FLD on CT imaging in an MDT setting.

## Background

Interstitial lung diseases (ILD) comprise the spectrum of disorders that affect the connective tissue framework of the lungs, termed the pulmonary interstitium. Whilst there are many non-fibrotic ILDs, there are essentially eight FLDs which will be described in this review (Table 1). The importance in ascertaining a diagnosis of an FLD is the fundamental desire to inform a patient of the nature of their disease, and its likely evolution over time. In this manner, a diagnosis informs prognosis, and simultaneously guides patient management decisions. The challenge faced when classifying FLDs lies with the variable prognoses implicit in the potential diagnoses. For example, a diagnosis of idiopathic pulmonary fibrosis (IPF) suggests a relentlessly progressive, aggressive disease where median survival might not extend beyond 5 years,^1^ yet fibrosis in the context of sarcoidosis can be associated with a more benign disease course.^2^

**Table 1.** The FLD

Fibrosing lung disease	Description
Chronic hypersensitivity pneumonitis	Long-term inflammation and fibrosis caused by an immune reaction to an allergen which may not be identified
Connective tissue disease-related interstitial lung disease	An assortment of diseases where FLD is diagnosed in conjunction with serology and clinical history
Cryptogenic organizing pneumonia	A clinical, histological and/or radiological appearance of inflammation and fibrosis secondary to lung injury of unknown cause
Drug-induced FLD	An uncommon form of FLD that results from toxic lung damage consequent to a variety of medications
Familial FLD	FLD presenting at a young age with heterogenous often cystic CT appearances
Idiopathic non-specific interstitial pneumonia	An increasingly uncommon entity following the change in IPF diagnostic guidelines.
IPF	An FLD of unknown cause, with a UIP picture on imaging and/or histology, with poor prognosis
Fibrotic sarcoidosis	A multisystemic granulomatous disorder of unknown aetiology, in which lung fibrosis occurs in a minority of cases

FLD, fibrosing lung diseases; IPF, idiopathic pulmonary fibrosis; UIP, usual interstitial pneumonia.

Whilst diagnosis initially required histopathological confirmation, advances in CT interpretation largely resulting from meticulous radiological–pathological correlation studies in the late 1980’s and early 1990s^3–6^ laid the foundations by which CT gained primacy in FLD diagnosis, as outlined in the 2011 IPF ATS/ERS/ALAT/JRS diagnostic guidelines.^7^ However, the limitations of CT when used alone in FLD diagnosis gradually became apparent as did the improved diagnostic concordance resulting from a case-based discussion incorporating a multidisciplinary team (MDT).^8^ Accordingly, a move towards a specialist MDT diagnosis became the new diagnostic gold-standard.^7,9^ Today, an MDT primarily comprises respiratory physicians, radiologists, and histopathologists, but with the involvement of rheumatological and allied health professionals when required.^10^

Whilst earlier IPF guidelines centered on reaching a single definitive diagnosis, the realization that FLDs are heterogenous and can evolve in a myriad of ways over time, has brought forward the concept of the “working diagnosis,” evaluating disease behavior over time.^11^ In the Fleischner Society diagnostic criteria for IPF,^12^ which expounds the use of a working diagnosis, a case reviewed at presentation is assigned the most probable diagnosis based on the available information at the time. However, the case is revaluated at a subsequent MDT six to twelve months later (or sooner should the patients conditions deteriorate acutely) and using clinical and radiological information indicating the aggressiveness of the disease, the diagnostic label may be modified. A recently developed diagnostic ontology for FLD, assigns cases discussed in an MDT a measure of diagnostic certainty, aiding the development of a single working diagnosis and allowing its evolution over time.^13^ In MDT discussions, CT appearances and evolution provide a large part of the evidence steering an MDT towards a working diagnosis.

With the recent publication of the 2018 update of the ATS/ERS/JRS/ALAT Clinical Practice Guidelines for diagnosing idiopathic pulmonary fibrosis (IPF),^11^ and the Fleischner Society diagnostic criteria for IPF,^12^ it is an opportune moment to review the spectrum of FLD and specifically the discriminatory power of CT for FLD identification and classification. By reviewing recent evidence and recommendations, this review aims to facilitate diagnosis and help guide patient management.

## CT scanning protocols

Given the importance associated with correctly diagnosing a FLD, it is imperative to obtain the best possible CT imaging in an individual patient. Current best practice guidelines for CT acquisition have evolved to reflect technological advances that allow improved spatial resolution of CT imaging. The latest consensus recommendations stipulate that all patients with a FLD should be imaged supine, at full inspiration using non-contrast enhanced volumetric CT imaging with a collimation <1 mm.^11^ Furthermore, expiratory CT imaging should be performed routinely to emphasize air-trapping, although expiratory imaging can be volumetric or interspaced.^11^

Should a contrast-enhanced CT be necessary, a non-contrast enhanced CT is recommended (either volumetric or interspaced) during the same examination. The paired non-contrast study allows distinction of ground glass density arising in the lungs secondary to contrast material, from genuine pathological damage to the lungs resulting in ground glass changes. Prone CTs are still recommended for their utility in distinguishing atelectasis from genuine subtle/early disease. However, new dose reduction techniques such as iterative reconstruction and tube current modulation have allowed the acquisition of low-dose CTs (1–3 mSv) which are diagnostically comparable to routine dose CTs in FLD.^11^ CTs performed at ultra-low dose (<1 mSv), however, are not recommended for routine use in the work-up of FLD patients.^11^

## Classification of fibrosing lung disease on CT

**Figure 1.**
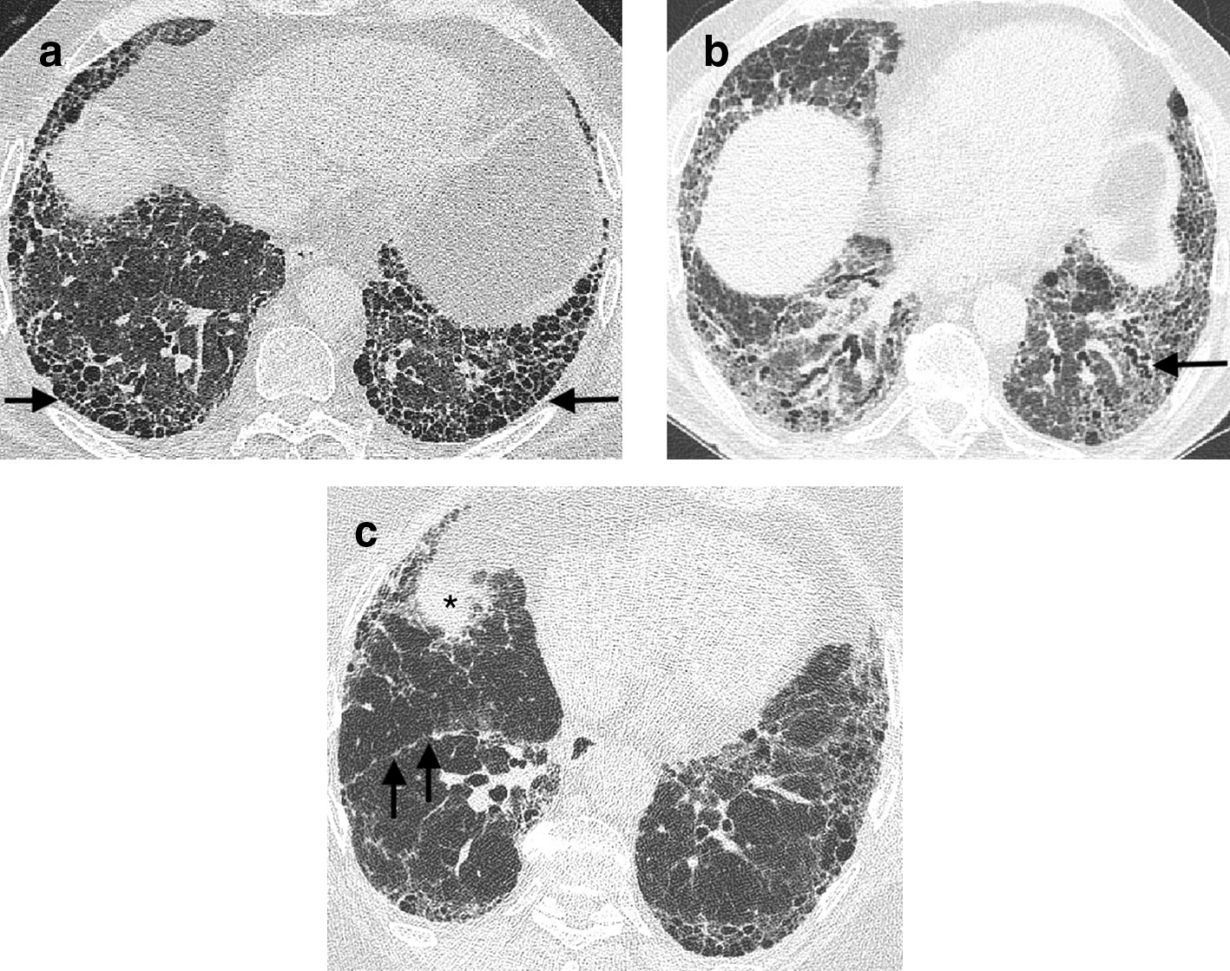
CT signs of lung fibrosis. Honeycomb cysts (arrow) lying in layers in the peripheral basal aspect of the lower lobes (a). Dilated varicose tortuous airways representing traction bronchiectasis (arrow) lying amidst dense fibrotic lung in the lower lobes (b). Volume loss in the lower lobes in a patient with idiopathic pulmonary fibrosis (c). In health, on axial CT images the most inferior aspect of the oblique fissures should reach the anterior chest wall at the level of the hemidiaphragms. However, in this patient, the right oblique fissure (arrows) has been pulled back and now only reaches the midpoint of the lung at a level where the right hemidiaphragm is visible (asterix).

There are three key features that suggest the presence of a FLD on HRCT: honeycomb cysts, traction bronchiectasis and volume loss. Honeycomb cysts refers to the presence of clustered, well-defined cystic spaces 3–10 mm in size (Figure 1a), which are typically in a peripheral, subpleural, and basal distribution.^14,15^ Destructive, paraseptal emphysema may lead to similar lung appearances, so close scrutiny of the morphology and distribution of the low attenuation regions, is key.^16^ For example, an absence of upper lobe emphysematous damage increases the likelihood that lower lobes cysts represent honeycombing over emphysema.

Interstitial fibrosis causes traction bronchiectasis by inducing contraction of the connective tissue scaffold around the airways (Figure 1b), leading to varicose dilation of the bronchi and bronchioles.^17^ A key discriminating feature of traction bronchiectasis, when compared to other causes of bronchial dilatation, is its distribution. Traction bronchiectasis is typically found peripherally, where the underlying supportive structure of the lungs is less strong, and airways are therefore more susceptible to deformation. Traction bronchiectasis should be accompanied by evidence of underlying causative fibrosis, either as reticulation or as ground glass opacification surrounding the dilated airway.^18^ Traction bronchiectasis may mimic the appearance of honeycombing, if severe, but viewing the CT in coronal or sagittal planes should delineate the tubular nature of the dilated airway.^19^ Rarely, an ongoing insult that results in interstitial damage can result in traction bronchiectasis. Yet, the traction bronchiectasis may resolve once the antagonizing stimulus is removed. This is most frequently reported in patients exposed to nitrofurantoin and emphasizes the utility of assigning a “working diagnosis” FLDs, where the underlying diagnosis can be informed by monitoring disease behavior over time.

The final key feature of fibrosis on HRCT is volume loss. In IPF, volume loss is predominantly lower zone and often asymmetrical,^20^ making it easy to detect by comparing the right and left oblique fissures’ relative positions. In health, the anterior aspect of the oblique fissures should nearly reach the anterior chest wall at the level of the hemidiaphragms,^18^ but the fissures are retracted when the lower lobes shrinks secondary to fibrosis (Figure 1c). While volume loss is the least specific of the three key signs of FLD, it can be useful for understanding the distribution of disease, and whether or not FLD may be present in cases where honeycombing and traction bronchiectasis are not obvious.

## UIP in IPF

Given the prognostic and management implications of a diagnosis of IPF, CTs of patients with FLD are primarily characterized according to how closely they mimic disease patterns and distributions classically seen in patients with IPF. A CT UIP pattern commensurate with a clinical diagnosis of IPF first needs to be ruled out before other CT diagnoses can be considered. Therefore, whilst the end-stage of most FLDs can manifest with a UIP pattern on CT, reasons for why the CT may reflect an alternative diagnosis need to be actively sought by the radiologist.

**Figure 2.**
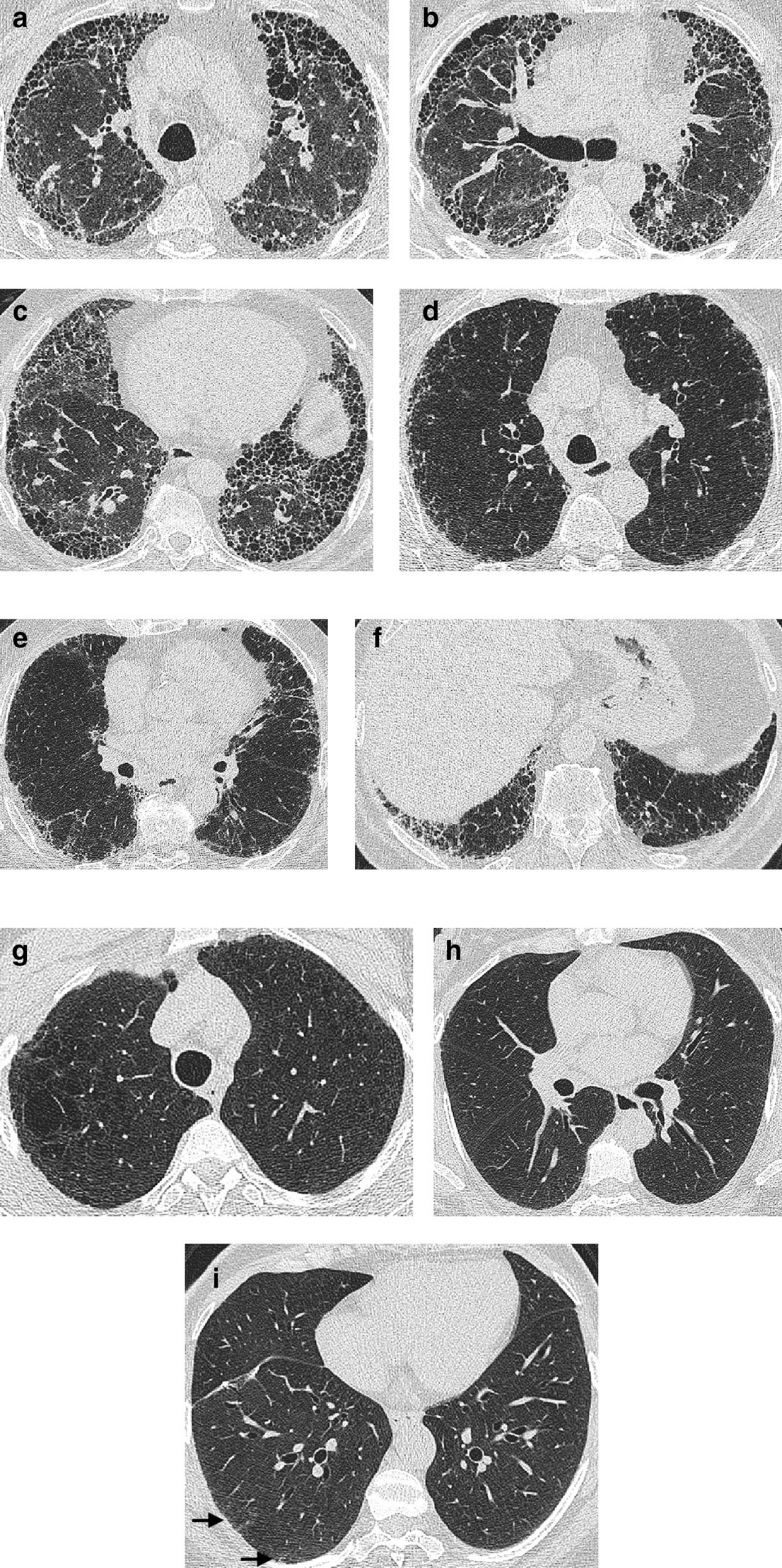
Images of the upper, middle and lower zones of the lung in a patient with a CT pattern of UIP (a–c), probable UIP (d–f) and indeterminate for UIP (g–i). A UIP pattern is characterized by a peripheral basal predominant distribution of disease with honeycombing, with no features suggestive of an alternative diagnosis. A probable UIP pattern is characterized by a peripheral basal predominant distribution of disease with traction bronchiectasis and no honeycombing, and no features suggestive of an alternative diagnosis. Subtle reticulation (arrows) in the right lower lobe and no associated traction bronchiectasis suggests a CT pattern indeterminate for UIP (i). UIP, usual interstitial pneumonia.

In essence, a CT demonstrating honeycombing with or without traction bronchiectasis in a subpleural and basal-predominant distribution (Figure 2a–c) and not exhibiting features suggestive of an alternative diagnosis is highly correlated to an underlying histological diagnosis of IPF.^1^ If honeycombing is present, but in an abnormal (*i.e.,* non-basal) distribution, consideration should be given to an alternative FLD diagnosis.

An CT pattern of probable UIP can be most easily thought of as analogous to a UIP pattern but where traction bronchiectasis is present alone without honeycombing (Figure 2d–f). Again, disease should predominate in a peripheral, subpleural, and basal distribution, and CT features suggestive of an alternative diagnosis should be absent. A “Probable UIP” pattern had been labelled a “Possible UIP” pattern in the earlier iteration of the Consensus IPF Guidelines.^7^ However, the realization that patients with a “Possible UIP” pattern often had an 82–94% likelihood of UIP being diagnosed on histology,^21,22^ enhanced the prognostic importance of a possible UIP pattern, especially in older, male ex-smokers with idiopathic disease.^23,24^

Scans that are indeterminate for UIP are those that do not fulfill the UIP or probable UIP pattern, and predominantly include CTs which demonstrate reticulation alone without honeycombing or traction bronchiectasis (Figure 2g–i). The cohort of patients with subtle CT changes or interstitial lung abnormalities that may or may not represent genuine disease is set to increase exponentially with the advent of lung cancer screening which will target patients (older heavy smokers) who are at high risk for developing FLD.^25,26^ Prone imaging might be necessary to confirm the presence of genuine fibrosis as distinct from dependent atelectasis. The other group of patients that may have scans indeterminate for UIP are patients that do not manifest a basal predominant distribution of fibrosis. Approximately, 30% of patients with an indeterminate UIP pattern are found to have a histological pattern of IPF,^27^ and accordingly the 2018 iteration of the consensus clinical practice guidelines suggest further diagnostic evaluation in this group with a surgical lung biopsy.^11^

CT scans that contain patterns indicative of fibrosis but which exhibit additional CT features (including cysts, mosaic attenuation, multiple nodules, predominant ground glass, consolidation and non-basal predominant distributions of disease) suggestive of an alternative diagnosis will be discussed in the following sections. Two “alternative CT features” have provoked consternation over the years and merit elucidation.

**Figure 3.**
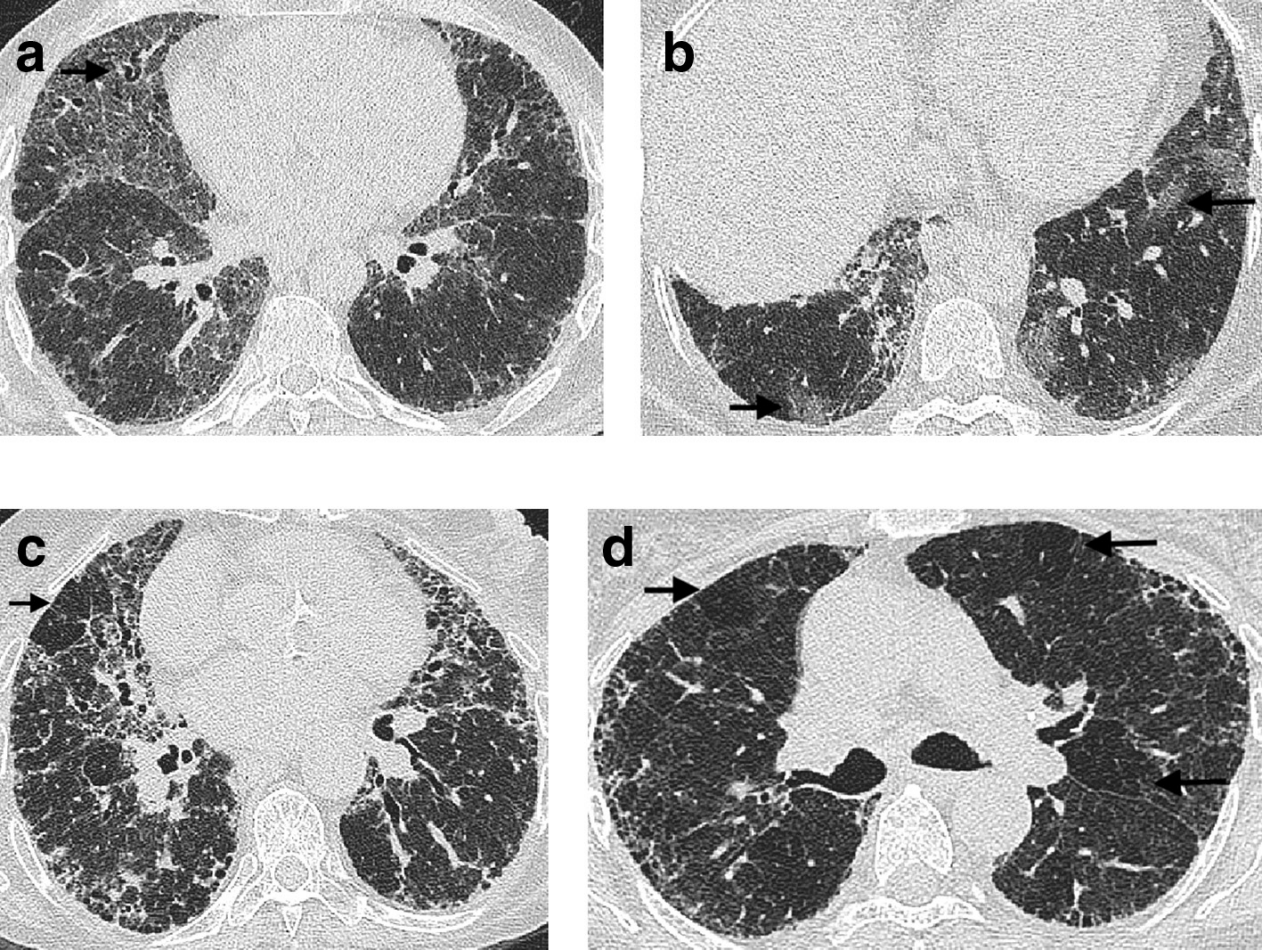
Axial CT image demonstrating ground glass density lying within areas of overlying reticulation and traction bronchiectasis (arrow) highly suggestive of fine fibrosis (a). When ground glass density lies separate to areas of fibrosis (arrow), it is far more likely to represent inflammation (b). Similarly, the low attenuation component of a mosaic attenuation pattern lying predominantly within fibrotic lung (arrow) is not specific for a diagnosis (c), but when it occurs in normal regions of lung (arrows), a diagnosis of chronic hypersensitivity pneumonitis can be made with greater confidence (d).

For many years, the presence of ground glass density on CT was synonymous with inflammation as it was thought to represent fluid infiltration within the airspaces.^14,28–30^ Subsequent nuanced, predominantly longitudinal studies demonstrated that ground glass densities could represent either fine fibrosis beyond the resolution of CT or inflammation that could respond to therapy.^31–33^ A consequence of this uncertainty was that ground glass densities that were extensive on CT (more than the extent of reticulation) were incompatible with a UIP/IPF pattern on CT in the 2011 IPF guidelines.^7^ The current guidelines stipulate that if ground glass densities predominate on a CT one should consider an alternative diagnosis where inflammation is common. However a useful imaging caveat, highlighted by the Fleischner guidelines,^12^ distinguishes ground glass densities overlying fibrotic lung (Figure 3a), which frequently reflect fine fibrosis, from ground glass densities occurring separate to fibrotic lung (Figure 3b). When geographically distinct from lung containing reticulation and traction bronchiectasis, there is greater certainty that ground glass densities represent inflammation. When such isolated ground glass areas are extensive, the possibility of an acute exacerbation should be considered.^34,35^

The second CT feature suggestive of an alternative diagnosis but which requires interrogation is the low attenuation component of a mosaic attenuation pattern. A mosaic attenuation pattern has invariably been associated with a diagnosis of chronic hypersensitivity pneumonitis where an inflammatory infiltrate can result in ground glass densities and granulomas can compress airway walls resulting in air-trapping.^36–38^ Yet, whilst air-trapping is a useful sign to distinguish CHP and IPF,^39,40^ air-trapping can also occur in IPF. Low attenuation lobules occurring in IPF (Figure 3c) invariably occur within regions of lung fibrosis^12^ and accordingly, a useful discriminator for CHP is the occurrence of spared pulmonary lobules (Figure 3d) within preserved normal lung parenchyma.^12^ Yet as fibrosis increases in extent, normal areas of lung become less frequent and distinguishing CHP from IPF becomes more challenging. In such cases, referring back to the patients earliest CT when disease was at its least extensive might prove beneficial.

## Nodular ossification and PPFE

**Figure 4.**
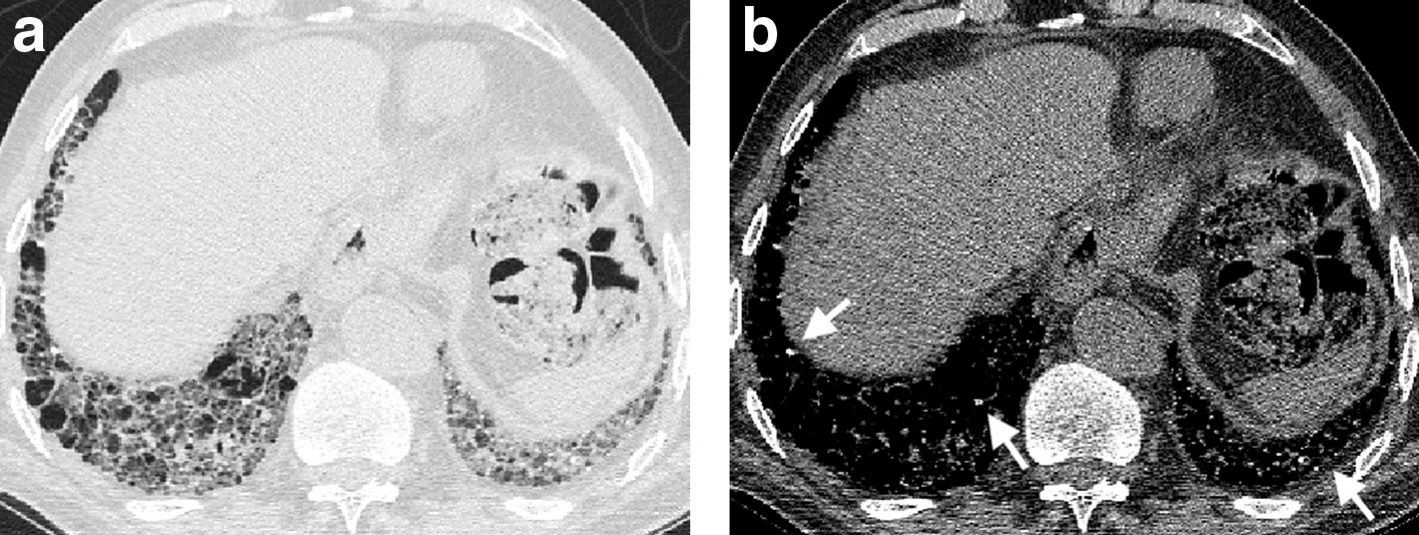
Nodular areas of parenchymal ossification (arrows) seen on axial CT images in the right lower lobe in a patient with idiopathic pulmonary fibrosis. Images are shown on lung windows (a) and mediastinal windows (b).

Two patterns that have been long identified as occurring in the context of FLD have also be emphasized in the 2018 IPF ATS/ERS/JRS/ALAT clinical practice guidelines.^11^ Nodular ossification reflects calcific deposition amongst regions of fibrotic lung and has been shown to preferentially occur in IPF (Figure 4) when compared to CHP and connective tissue disease-related ILD (CTD-ILD).^41^ Nodular ossification has also been found to be more common in cases of UIP than NSIP,^41^ and therefore may act as a diagnostic aid, enhancing the confidence with which a UIP pattern is ascribed to a CT.

**Figure 5.**
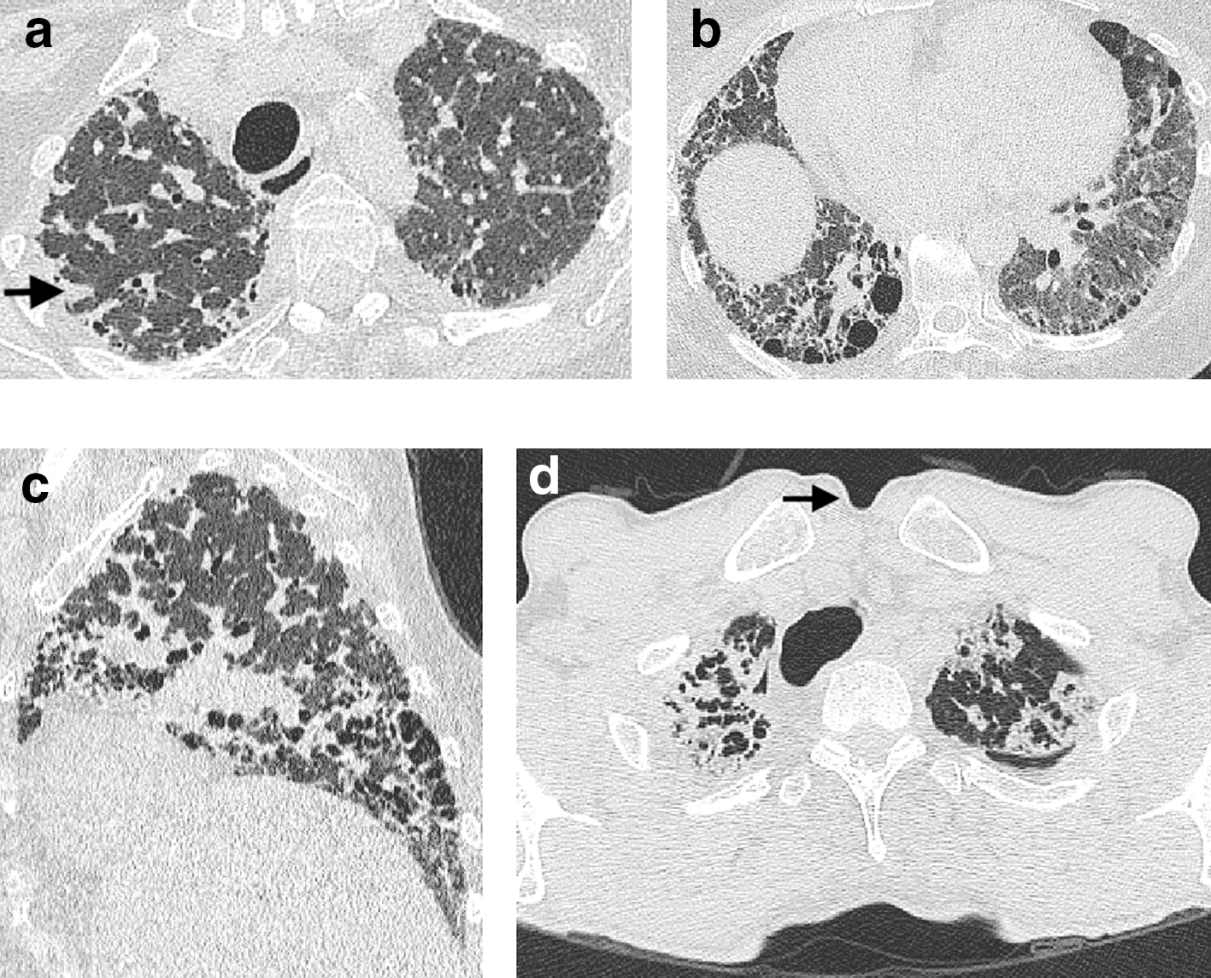
CT features of pleuroparenchymal fibroelastosis in a patient with idiopathic pulmonary fibrosis. Pleuroparenchymal fibroelastosis is characterized by pleurally based aggregations (arrow) of dense connective tissue in the lung apices (a). Honeycomb cysts in keeping with the underlying idiopathic pulmonary fibrosis diagnosis are evident at the lung bases (b + c). A suprasternal depression (arrow) and bronchiectasis in the upper lobes are also frequently associated with pleuroparenchymal fibroelastosis (d).

Pleuroparenchymal fibroelastosis (PPFE) has been recognized as an idiopathic condition,^42–44^ or one associated with graft *vs* host disease in the context of organ transplantation^45^ for several decades. But an increased recognition of the entity in the context of a variety of FLDs^46^ resulted in PPFE being included in the consensus classification of the idiopathic interstitial pneumonias in 2013.^47^ PPFE presents as aggregations of connective tissue which are often angular in shape and pleurally based (Figure 5a–c) and which predominate in the lung apices.^44^ Associated imaging features include a flattened anteroposterior thoracic diameter,^48^ a supraclavicular depression of the anterior chest wall^49^ (Figure 5d), and an increased incidence of upper lobe bronchiectasis (Figure 5a) and pneumothoraces.^46,49,50^ The 2018 consensus clinical practice guidelines^11^ advise reporting a CT with evidence of PPFE and FLD in the context of a UIP pattern. Until more is learnt about the superadded functional and mortality effects resulting from co-existing PPFE, diagnosis and patient management will remain centered on the FLD itself.

### UIP in other FLDs

**Figure 6.**
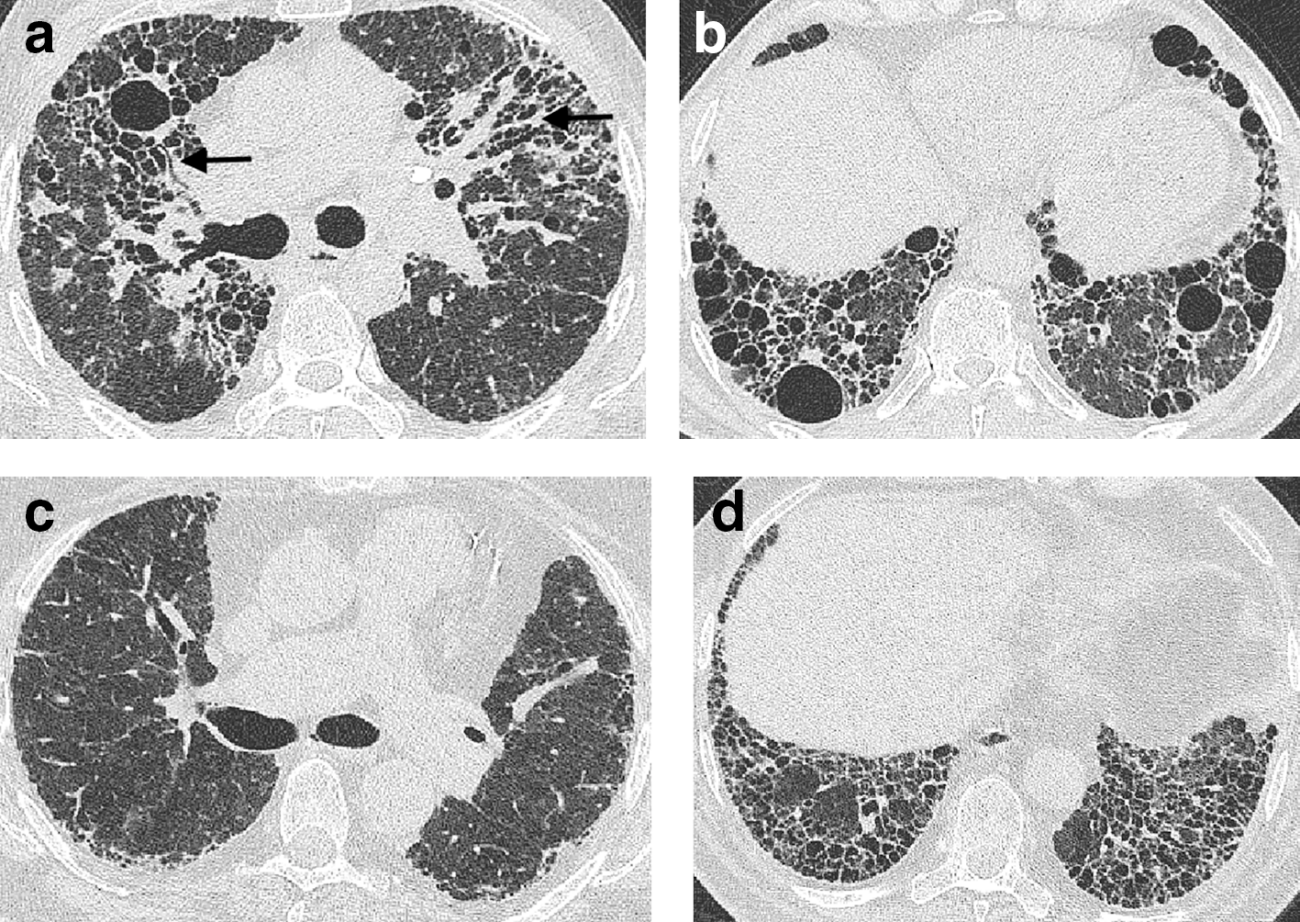
A usual interstitial pneumonia pattern in a patient with chronic hypersensitivity pneumonitis, characterized by honeycomb cysts in the lung midzones (a) and more extensive cysts in the lower zones (b). The fibrosis in the midzones demonstrates a bronchocentric distribution (arrows) which is associated with chronic hypersensitivity pneumonitis (a). A usual interstitial pneumonia pattern can also occur in a patient with rheumatoid arthritis-related interstitial lung disease. Peripheral traction bronchiectasis is seen in the upper zones (c) and honeycombing is evident in the lower lobes (d).

As mentioned earlier, a confusing aspect in the study of the FLDs has been the conflation of a UIP pattern with a diagnosis of IPF. A UIP pattern on CT is a morphological pattern that reliably reflects histopathological features on a surgical lung biopsy.^1,51–55^ A UIP pattern therefore necessitates clinical confirmation of an idiopathic cause to the patients condition for an IPF diagnosis to be made. Accordingly, it is unfortunate that the terms UIP and IPF are often used interchangeably when in reality not infrequently, patients with CHP^39,56–58^ (Figure 6a + b) and rheumatoid arthritis-related interstitial lung disease (RAILD)^59–61^ (Figure 6c + d) and rarely sarcoidosis^62^ have honeycombing identified on CT.

Numerous studies have demonstrated that when honeycombing occurs in patients with CHP^58^ or RAILD^59–61^ patients outcomes are similar to those of IPF patients. At present given the differences in management between IPF and other FLDs, distinguishing CHP and RAILD from IPF with a UIP pattern remains important. However, ongoing clinical trials evaluating antifibrotic medication in patients with progressive fibrotic phenotypes^63,64^ may coalesce management strategies regardless of aetiology in patients with a CT UIP pattern, rendering diagnostic distinctions less important.

### NSIP pattern

An NSIP pattern on CT represented one of the cardinal patterns of idiopathic interstitial pneumonia in early iterations of consensus guidelines.^65^ Though the 2013 update to the consensus guidelines^47^ retains NSIP as a key CT pattern, outside the confines of a CTD-ILD or CHP, NSIP is infrequently identified on CT and diagnoses of idiopathic NSIP are rarely made in MDT settings. Though NSIP is a common pattern in CHP, accessory CT features such as a bronchocentric distribution of fibrosis, an upper lobe predominance of disease and mosaic attenuation imply a diagnosis of CHP over a pattern of NSIP.

The change in diagnostic frequency of NSIP has primarily been a consequence of the 2011 ATS/ERS/JRS/ALAT IPF diagnostic guidelines^7^ which required the interrogation of CTs through the prism of UIP. CTs without honeycombing but where traction bronchiectasis was evident in a basal predominant, peripheral distribution and which in 2009 would have been classified as NSIP, were now given the label of possible UIP. Furthermore, the recognition that idiopathic NSIP might be a precursor of an as yet undeclared CTD-ILD has brought about terms such as undifferentiated CTD^66^ and interstitial pneumonia with autoimmune features (IPAF).^67^ Though these are yet to become established as formal diagnoses, they have reduced the proportion of MDT cases confidently diagnosed as idiopathic NSIP.

**Figure 7.**
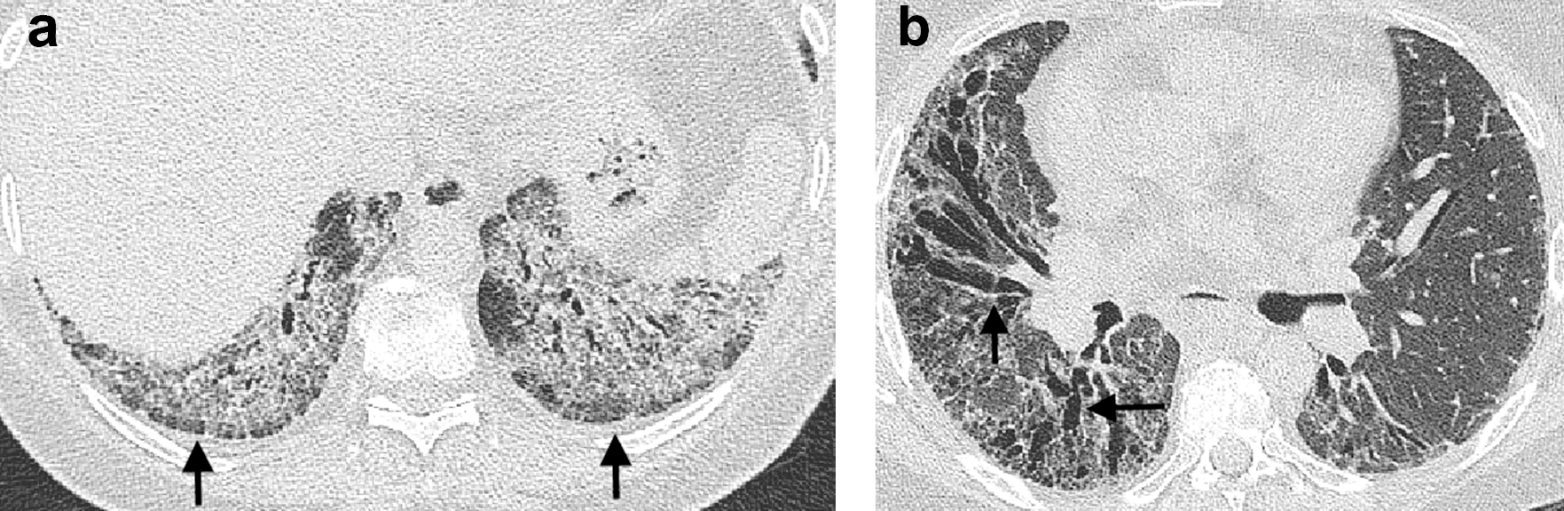
Subpleural sparing of fibrosis (arrows) in a patient with non-specific interstitial pneumonia (a). A patient with a non-specific interstitial pneumonia pattern (b) where traction bronchiectasis (arrows) is distributed more centrally than is typically seen in a usual interstitial pneumonia pattern.

An NSIP pattern has been associated with more central traction bronchiectasis (Figure 7a) when compared to UIP where traction bronchiectasis occurs more peripherally in the lung.^68^ Subpleural sparing has been recognized as a hallmark of NSIP (Figure 7b) which is rarely seen in UIP^39^ and a more regular pattern to reticular lines has been suggested in NSIP, compared to irregularly spaced lines of varying thickness in UIP.^12^ When diagnosed in an MDT setting NSIP is often a working diagnosis demanding review at subsequent MDTs given the potential for the development of a CTD or the longitudinal evolution of an NSIP pattern to a UIP pattern on serial CT examination.^69^

## CHP vs IPF

**Figure 8.**
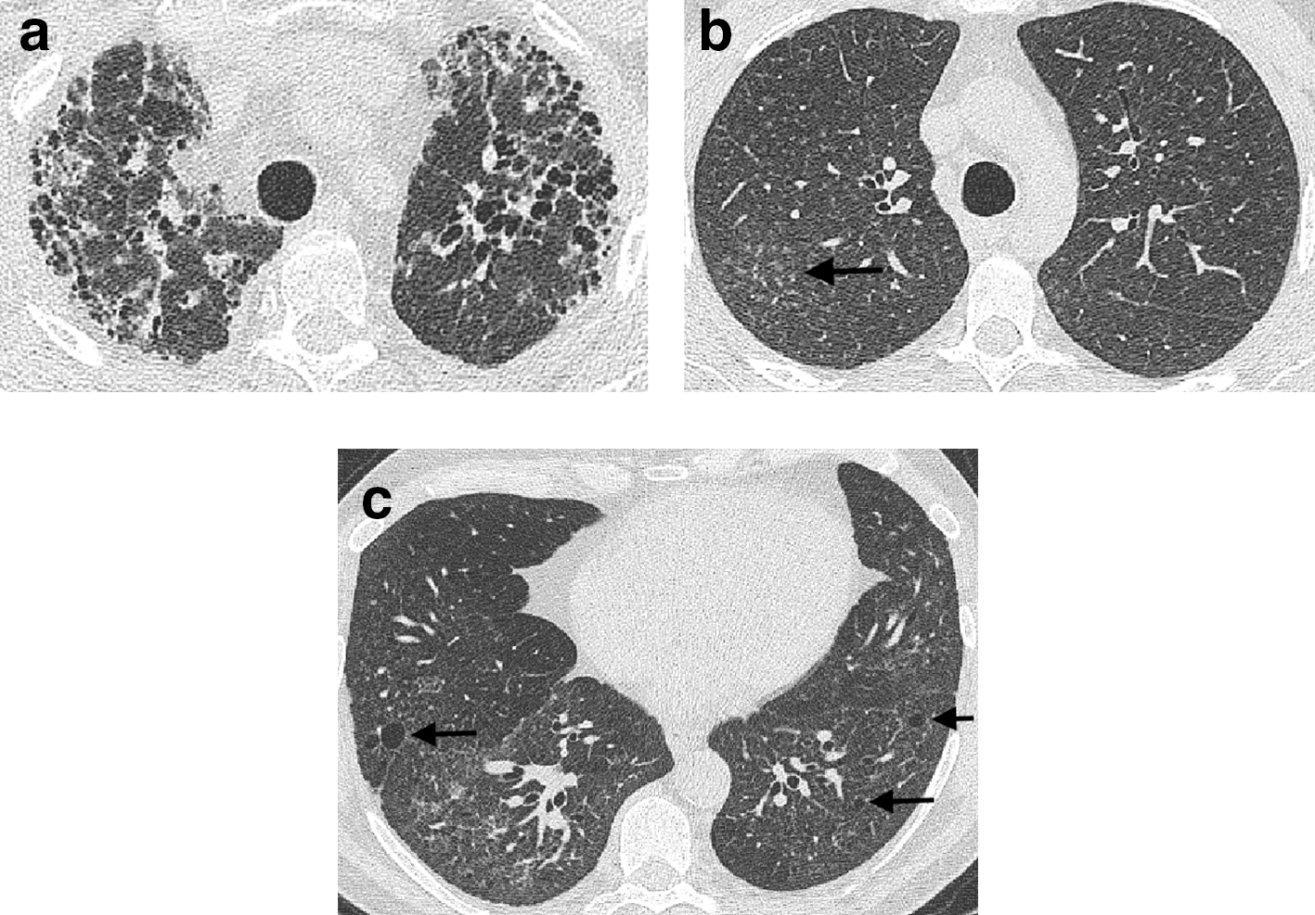
A bronchocentric distribution of fibrosis in a patient with chronic hypersensitivity pneumonitis (a). Centrilobular nodules (b) and pulmonary cysts (c) are seen in other examples of chronic hypersensitivity pneumonitis (arrows).

The most common diagnostic dilemma faced by an MDT is distinguishing CHP from IPF. CHP can be upper lobe predominant, diffusely distributed throughout the lung, or lower zone predominant in one-third of cases.^39^ The combination of an axially diffuse distribution of disease and more low attenuation lung (representing the low attenuation component of a mosaic attenuation pattern) than reticulation on CT was recently associated with a high specificity low false diagnosis risk for CHP.^40^ With regard to other CT features that might suggest CHP, a bronchocentric pattern to fibrosis (Figure 8a) can be a subtle sign that is far harder to identify than the well-described observation of nodules distributed in a centrilobular pattern (Figure 8b) or the presence of cysts (Figure 8c).

In practice, making a diagnosis of CHP has been handicapped by the lack of consensus diagnostic guidelines^70,71^ that over time has helped refine the diagnosis of IPF. The requirement for clear diagnostic guidelines in CHP is especially pertinent as clinical criteria such as evidence of antigen exposure may be lacking in up to 60% of cases^72,73^ and the diagnostic value of serum precipitants remains unclear.^12^ Furthermore, CT analysis forms just one aspect of the diagnostic pathway in CHP as bronchoalveolar lavage differentials can strongly contribute to diagnostic certainty. Yet, here again there is as yet no consensus on the level of lymphocytosis required to make a high likelihood diagnosis of CHP.^70,74^ Until consensus guidelines for a CHP diagnosis emerge, MDT meetings will continue to focus heavily on trying to disentangle cases of CHP and IPF, some of which will be labelled as unclassifiable FLD.

## Unclassifiable FLD

The spectrum of unclassifiable FLD comprises a heterogenous assortment of patients which constitute between 10 and 25% of all ILD cases.^75–78^ Patients can be labelled unclassifiable FLD if there is no clear first-choice diagnosis that can be made with over 50% certainty despite evaluation of all available data, or if no clear diagnosis can be made following a surgical lung biopsy.^13^ Reaching a clear diagnosis on an individual case is also in part determined by the proclivities of an individual clinician. For example, a fastidious clinician might require that all diagnostic criterion need to be satisfied before advocating a single diagnosis, whilst another clinician might take a more pragmatic approach and manage a patient based on the most likely diagnosis given the available data. As the number of patients undergoing surgical lung biopsies reduces and whilst several FLDs lack diagnostic guidelines, diagnoses are often made on the basis of likelihoods.

**Figure 9.**
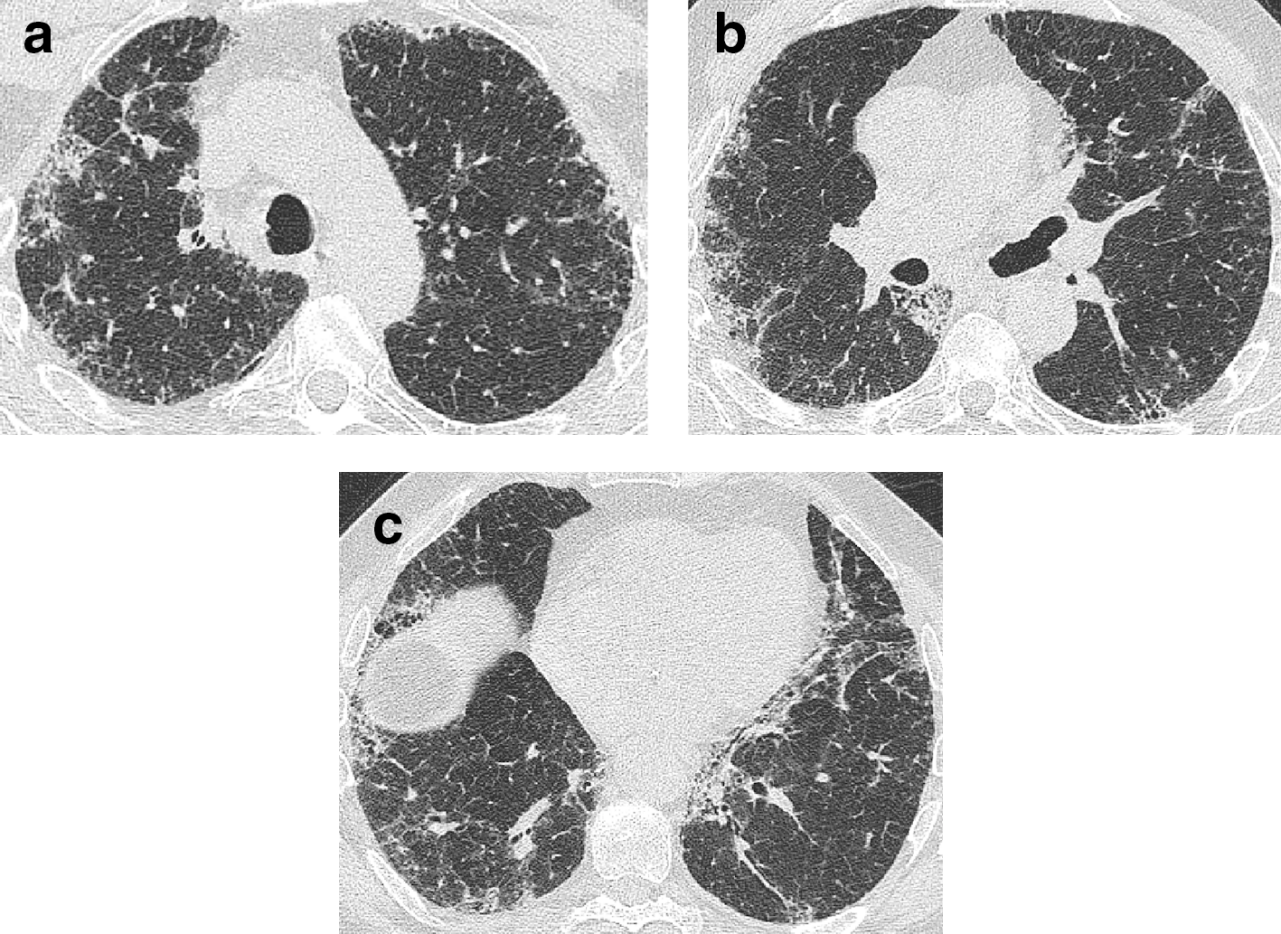
A diffuse distribution of fibrosis throughout the upper (a), middle (b) and lower zones (c) of the lungs in a patient diagnosed as unclassifiable fibrosing lung disease. There is no peripheral, basal-predominant distribution of disease as is commonly seen in a usual interstitial pneumonia pattern occurring in idiopathic pulmonary fibrosis.

Unclassifiable FLD is the exemplar condition for which the concept of a working diagnosis of a FLD could be employed.^12^ Here, given that there is no clear diagnosis, patient could be managed on the basis of a working diagnosis, with annual or biannual reviews of the clinical picture in future MDTs. Given that the CT will invariably exhibit features suggesting alternate patterns to UIP (Figure 9a–c), regular CT reviews will be essential both to monitor the evolution of CT patterns, and provide some statement on disease trajectory.

## Fibrotic sarcoidosis

**Figure 10.**
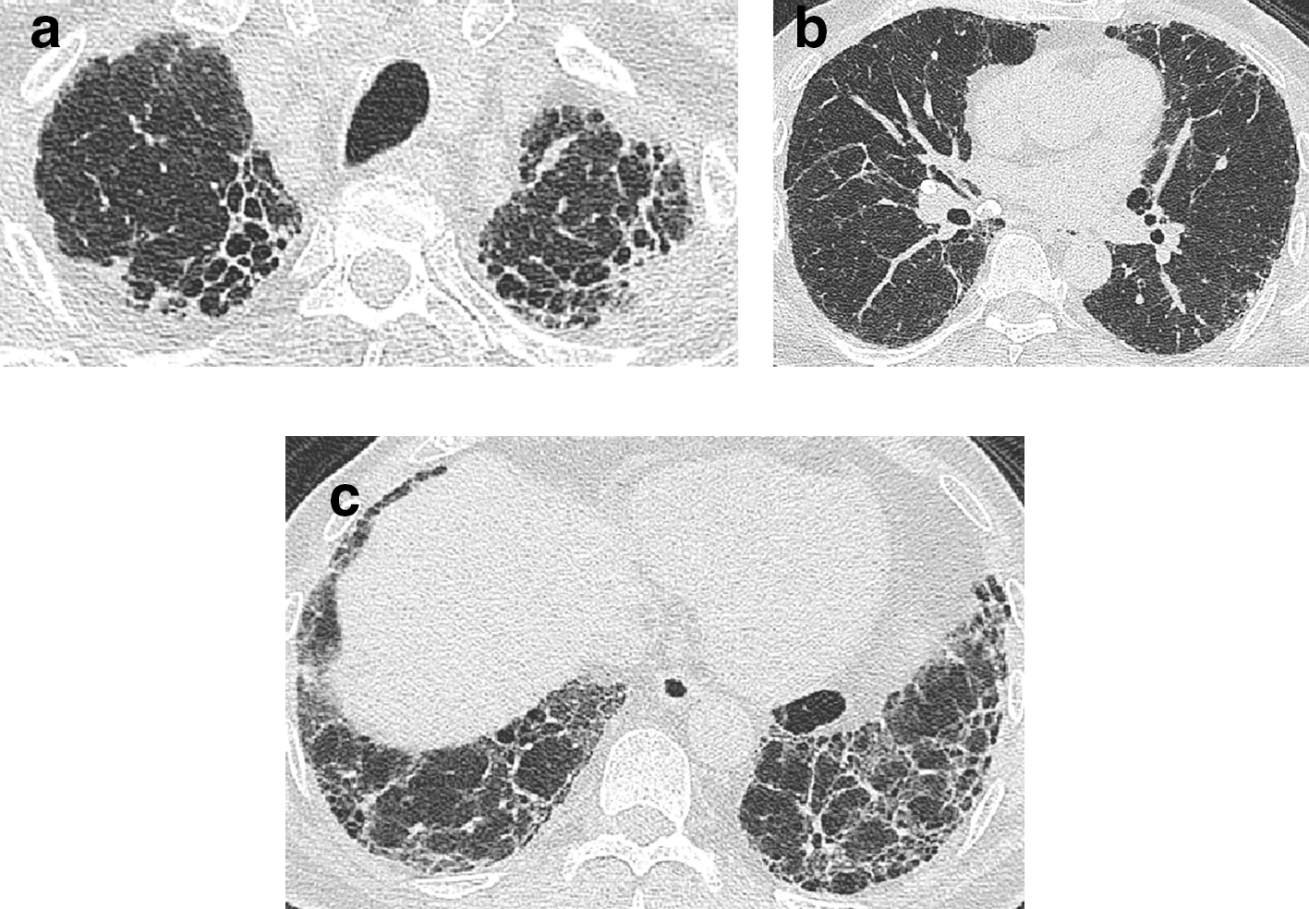
Upper zone fibrosis (a) affecting the posterior aspects of the upper lobes in a patient with fibrotic sarcoidosis. Disease is limited in the middle zones (b), but honeycomb cysts are visible in the lower lobes in keeping with a usual interstitial pneumonia pattern (c). Note is also made of calcified mediastinal nodes on the right which are frequently seen in patients with sarcoidosis.

When fibrotic, sarcoidosis results in a pattern of fibrosis that radiates from the hilar regions of the lung and extends into the posterior aspects of the upper lobes (Figure 10a–c) resulting in severe architectural distortion.^79^ Volume loss and traction bronchiectasis are invariably seen, whilst honeycomb appearances in the form of apical bullous destruction admixed with destructive emphysema is not uncommon.^80^ The upper lobe predominance of sarcoidosis allows distinction from a UIP pattern associated with IPF but can make distinguishing sarcoidosis from CHP challenging. Sarcoidosis can occasionally be seen in the lower zones of the lungs, where honeycombing (suggesting a UIP pattern) or nodular thickened interlobular septa might be present. Yet the degree of damage in the upper lobes, as well as subtle nodularity within the lungs and enlarged, potentially calcified mediastinal and hilar nodes, might suggest the underlying diagnosis.

### CTD-ILD, familial and drug-induced FLD

**Figure 11.**
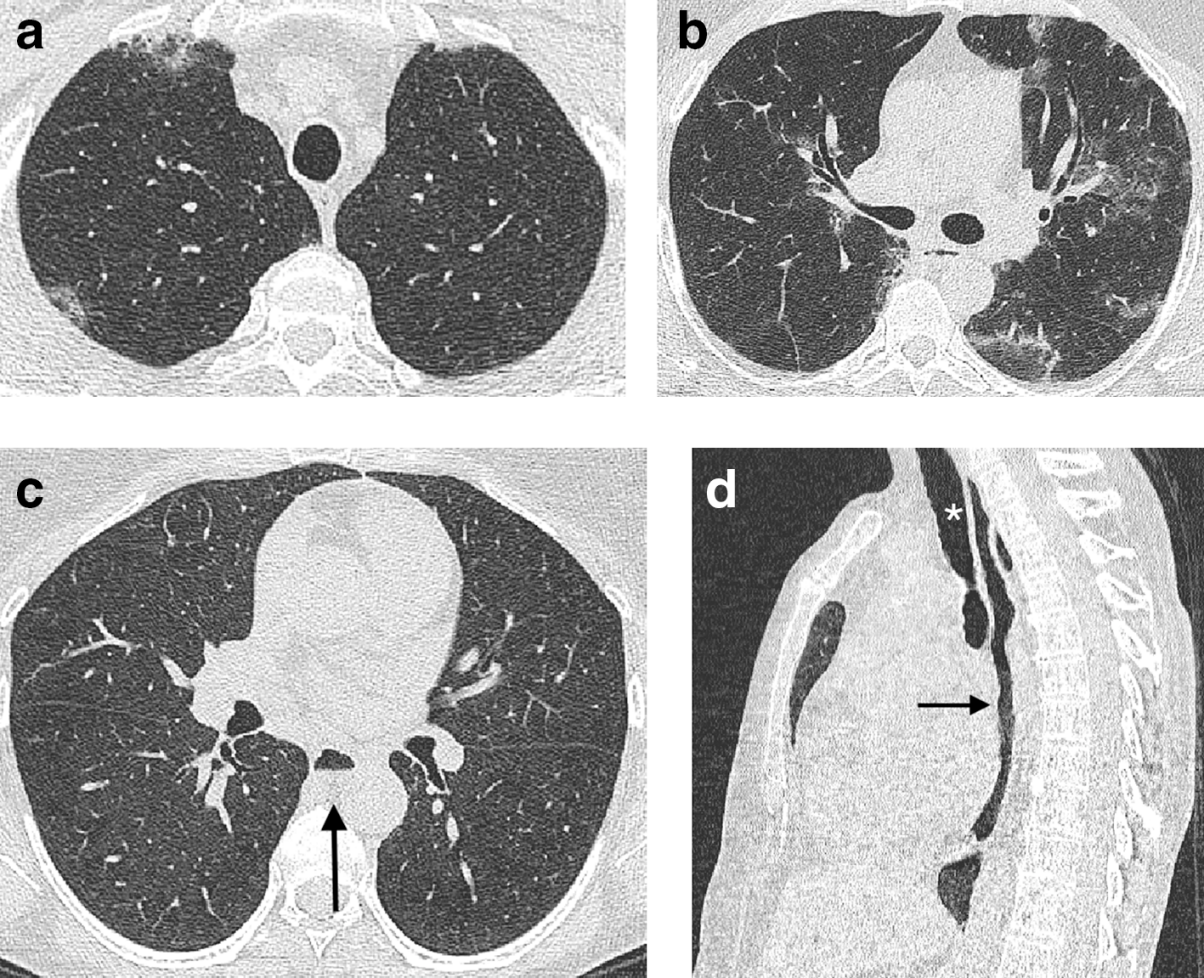
Organizing pneumonia in the upper (a) and middle (b) zones in a patient with polymyositis. The organizing pneumonia is predominantly located peripherally in the lung and is characterized by arcs of dense fibrosis surrounded by ground glass density in keeping with the reverse halo or Atoll sign. Organising pneumonia can demonstrate a wide variety of appearances on CT. A dilated patulous oesophagus (arrows) on axial (c) and sagittal (d) CT imaging in a patient with scleroderma. An air-fluid level is visible in the oesophagus on axial imaging; the trachea is demarcated with an Asterix.

The spectrum of CTD-ILD is beyond the scope of the current review but has been well described in prior reviews.^81^ In practice, diagnosing a FLD in the context of CTD-ILD is less of a clinical conundrum for the radiologist as the patient often presents with a CTD diagnosis or will be evaluated by a rheumatologist if the pulmonologist harbours any suspicion of a CTD. CTD-ILDs can manifest a broad array of CT appearances. These range from airways disease,^82,83^ UIP and NSIP in RAILD,^84,85^ to UIP and NSIP in scleroderma,^86–89^ NSIP and organizing pneumonia (Figure 11) in polymyositis and dermatomyositis^90,91^ and Sjogrens syndrome^92–94^ and airways disease and NSIP in mixed connective tissue disease.^95–97^ Occasionally, accessory signs on a CT such as a patulous dilated oesophagus as seen in patients with scleroderma may suggest a diagnosis (Figure 11).

Whilst it is increasingly apparent that cases of NSIP on CT might display clinical features suggestive of but not diagnostic of a CTD-ILD, until IPAF establishes itself as a formal diagnostic category,^67^ imaging clues that suggest the possibility of IPAF require consideration. These include evidence of multicompartmental disease on CT where airways disease, pleural disease and interstitial disease may all coexist. Patients may also display disproportionate pulmonary hypertension manifesting on CT as enlargement of the main pulmonary artery or an increase in the pulmonary artery aorta ratio. Recent reports in patients with IPAF features have demonstrated an IPF-like poor outcome when honeycombing has been identified on CT.^98^

Though also out of the scope of this review, diagnosing drug-induced and familial FLD relies on a high level of clinical suspicion and appropriate clinical histories. CT patterns of drug-induced FLD are most frequently those of organizing pneumonia and NSIP, with UIP almost never identified. CT appearances of familial FLD can be very heterogenous, but cystic changes in a young patient are suspicious for a surfactant deficiency disorder.^99^

### MDT input from radiology

When discussing a case from a radiological standpoint in an MDT setting, several key aspects need to be conveyed. Firstly, the CT should be reported in UIP terms and categorized as: UIP, probable UIP, indeterminate for UIP or potential alternative diagnoses proffered. If the CT is indeterminate for UIP, reasons for this should be given and further imaging suggested if required (*e.g.,* prone CTs).

**Figure 12.**
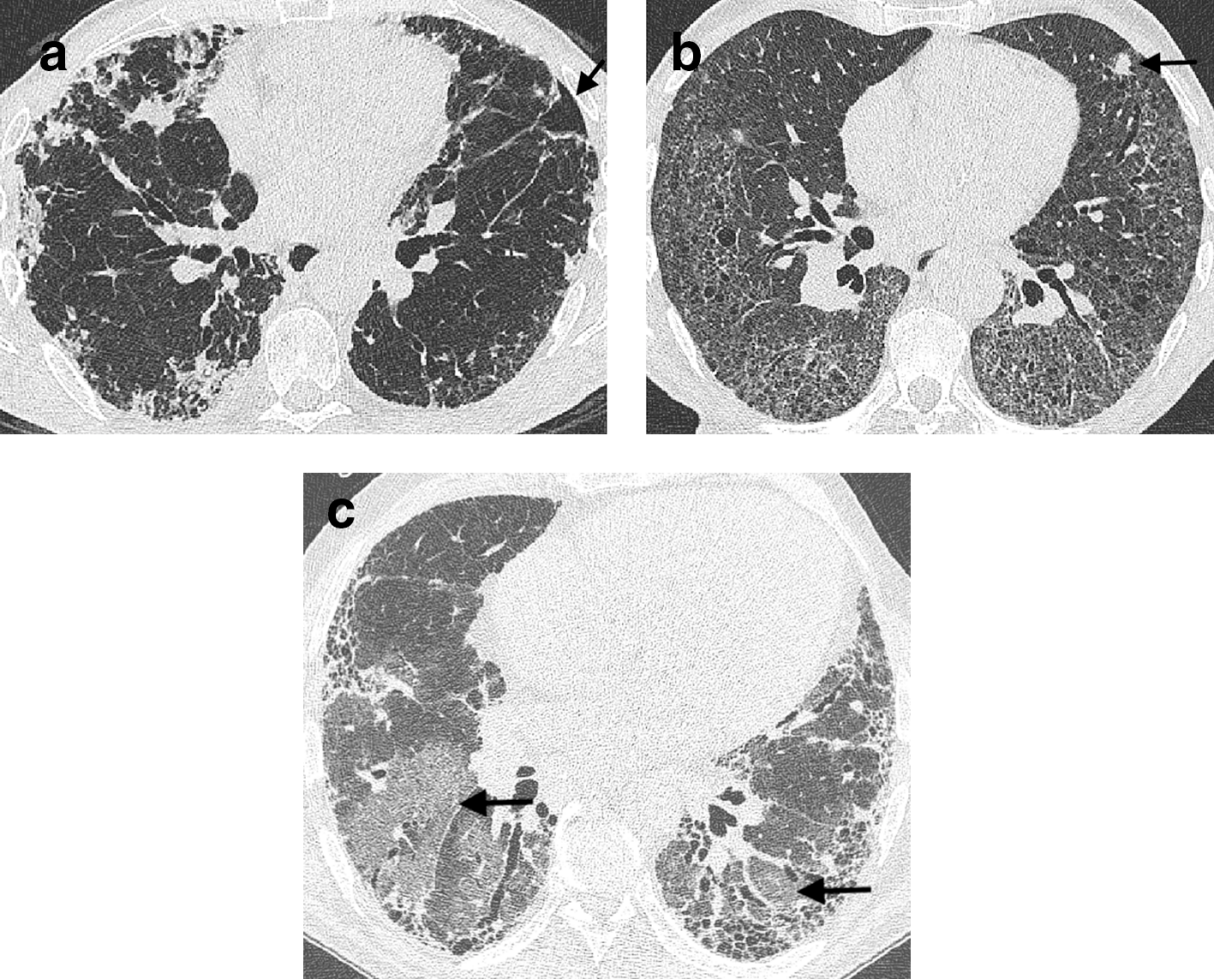
Important complications to identify on a CT in a patient with a fibrosing lung disease. A pneumothorax (a) can present as acute shortness of breath (arrow). Newly developing lung nodules (arrow) may represent early lung cancer which has a higher likelihood of developing, in patients with fibrosing lung disease (b). Widespread ground glass density occurring in normal regions of lung (arrows) should raise the possibility of an acute exacerbation of disease (c).

If an alternate diagnosis is suggested, reasons for this should be highlighted and the differentials listed. Unclassifiable FLD should be considered as a potential diagnosis. The differential diagnosis list that arises will help in the formulation of a working diagnosis and a degree of certainty should be ascribed to the working diagnosis. The presence of disease complications on a CT should always be highlighted. These include a pneumothorax (Figure 12a) or pneumomediastinum, discrete nodules which could represent an early lung cancer (Figure 12b), foci of ground glass in normal regions of lung that could represent infection, pulmonary oedema or an acute exacerbation (Figure 12c) if extensive and the size of the main pulmonary artery indicating the possibility of pulmonary hypertension.

Lastly, further investigations that might progress reaching a diagnosis should be suggested. Documentation of an MDT discussion is a crucial but often neglected step. The formal working diagnosis and the alternative diagnoses should all be listed. CT complications and alternative investigations required should be highlighted and a date marked at which the patient is due to be rediscussed in the MDT.

### The future

Despite the emergence of two diagnostic guidelines for the management of FLD in 2018,^11,12^ it is still possible that the diagnostic process for FLDs may change in the coming years. The main levers of change include the introduction of computer-based machine learning techniques for disease classification on CT^100^ which may help adjudicate challenging cases in expert centres or aid diagnosis in non-specialist centres. The results of clinical trials examining a progressive fibrotic phenotype of FLD^63^ may also simplify diagnosis as several conditions where fibrosis can be objectively shown to progress (identified using functional, symptomatic, exercise-related or computer-based imaging metrics) might have common management strategies. Lastly the development of guidelines for the diagnosis of CHP and the running of MDTs will standardize practice around the world and, it is hoped, introduce a common language to the evaluation of FLD.

## Conclusion

Diagnosing FLD on CT remains challenging, yet is crucial as it is the starting point for optimizing patient care. Whilst the requirement to arrive at a single final diagnosis following MDT discussion has lessened with the advent of a working diagnosis, diagnostic certainties should improve as guidelines for conditions such as CHP emerge. A small number of complex cases require a disproportionate amount of MDT discussion and in these difficult cases, continued review of patients in an MDT setting, which may in the future include geneticists, immunologists, palliative care specialists amongst others, will allow better patient specific care. As our knowledge of the mechanisms and subtypes of FLD grows, the diagnostic landscape will change appropriately. Until then, this review aims to disentangle and simplify the diagnostic approach to this complicated array of conditions.
